# Efficacy of *Myristica fragrans *and *Terminalia chebula* as Pulpotomy Agents in Primary Teeth: A Clinical Study

**DOI:** 10.5005/jp-journals-10005-1565

**Published:** 2018

**Authors:** Shikha Mali, Shilpy Singla, Arun Sharma, Ankit Gautam, Babita Niranjan, Shantanu Jain

**Affiliations:** 1-3 Department of Paedodontics and Preventive Dentistry, Peoples College of Dental Sciences and Research Centre, Bhopal, Madhya Pradesh, India; 4 Private Practitioner, Bhopal, Madhya Pradesh, India.; 5 Department of Pedodontics and Preventive Dentistry, Rishiraj College of Dental Sciences and Research, Bhopal, Madhya Pradesh, India.; 6 Department of Pedodontics and Preventive Dentistry, Mahatma Gandhi Dental College and Hospital, Jaipur, Rajasthan, India

**Keywords:** Formocresol, *Myristica fragrans*, Pulpotomy, Primary teeth, *Terminalia chebula*

## Abstract

**Background:**

Pulpotomy is the treatment for cariously exposed vital primary molars. UsingfFormocresol as a pulpotomy agent is been in controversy, which has triggered the search for better alternatives. The product like ‘Myristica fragrans (MF)–Nutmeg gel, *Terminalia chebula* (TC)–Myrobolan gel is gaining popularity as herbal pulpotomy agents.

**Aim:**

To evaluate and compare clinical and radiographical success of herbal gels *Myristica* fragrans (MF)–Nutmeg, and *Terminalia chebula* (TC)–Myrobolan as pulpotomy medicaments in primary teeth.

**Materials and methods:**

Twenty participants (n = 20), each with at least two primary molars requiring pulpotomy, were selected and divided into two test groups. In 10 children Terminalia chebula gel was placed in one side and Formocresol on another side. Rest 10 children were treated with Myristica fragrans gel on one side and another side with formocresol. The treated teeth selected for clinical and radiographic evaluation were monitored periodically for 3, 6 and 12 months.

**Results:**

With the follow-up of 12 months there was no significant difference in efficacy of all three pulpotomy medicaments, i.e. Nutmeg, Myrobolan, and Formocresol, respectively was found

**Conclusion:**

Herbal gels have a promising role in dentistry having the proper knowledge, and their effects on teeth would prove them as a successful dental therapeutic agent.

**How to cite this article:**

Mali S, Singla S, Sharma A, Gautam A, Niranjan B, Jain S. Efficacy of *Myristica fragrans* and *Terminalia chebula* as Pulpotomy Agents in Primary Teeth: A Clinical Study. Int J Clin Pediatr Dent, 2018;11(6):505-509

## INTRODUCTION

Devitalizing pulpotomy is the most common treatment procedure considered in primary teeth. The medicament used most frequently in pulpotomy and proven for long is formocresol (FC). It is considered despite controversial implications such as carcinogenic and mutagenic effects. This led to different alterations for pulpotomy agents that provide better clinical efficacy without secondary effects. Medicinal plant extracts have been effectively used in various dental treatments due to higher safety margins and lower costs.^1^

Studies have reported that *Myristica fragrans*–Nutmeg and *Terminalia chebula*–Myrobolan, have antimicrobial, anti-inflammatory and antioxidant properties.^2^ *Myristica fragrans* Houtt (Family: Myristicaceae) locally known as buah pala in Malay is mostly cultivated for spices. Nutmeg is the seed kernel inside the fruit and mace is the fleshy red, covering the kernel.^3^ *Myristica fragrans* have been found to be alkyl benzene derivatives (myristicin, elemicin, safrole, etc.).^4^ It has a strong antibacterial effect against oral microorganisms such as *Streptococcus* species, and *Lactobacillus* species, and also exhibiting some activity against *Actinomyces viscosus*, *Porphyromonas* *gingivalis,* and *Staphylococcus aureus.*^5^

*Terminila chebula* is a medicinal plant widely distributed in south Asian countries. It is an antioxidant with free radical scavenging activities.^6^ It also shows anticancer activity and against *Helicobactor pyroli*.^7^ Both aqueous and ethanolic extract of *T. chebula* has strong antimicrobial activity against the uropathogen and oral pathogens.^8^

Extracts of *T. chebula* showed antimicrobial activity against methicillin resistant *Staphylococcus* *aureus* and inhibitory action against *Salmonella typhi* and intestinal bacteria.^9^

Therefore this study aims at evaluating and comparing, clinical and radiographical efficacy of *Myristica fragrans* (Nutmeg), *Terminalia chebula* (Myrobolan) and formocresol, when used as pulpotomy agents following pulpotomy in human first and second primary molars.

## MATERIALS AND METHODS

The study was undertaken in the Department of Paedodontics and Preventive Dentistry. *Ethical clearance was obtained from the Institutional Review Board and informed written consent was obtained from the parents*.

### Preparation of Extracts

Seeds of nutmeg, and myrobolan were subjected to shade drying and grounded into fine powder. The 45 gm of powder was then subjected to Soxhlet extraction with 90% ethanol as solvent. Soxhletion process was allowed to carry out till the complete extraction of sample material used for extraction with the maintenance of temperature 70°C the boiling points of the solvents used. Post phytochemical extraction, it was transferred into clean and pre-weighed universal tubes. Then tubes were stored at 4–8°C in the refrigerator for 24 hours. Then percentage yield was calculated as dividing the initial weight of raw material with the final weight of extracts.

**Figure:**
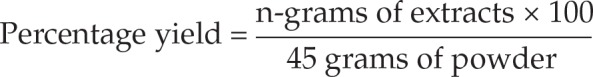


where n indicates the final weight of nutmeg and myrobolan extracts.

### Minimal Inhibitory Concentration Determination for Extracts

One gram of ethanolic extract of nutmeg and myrobolan were dissolved in 10 mL of distilled water separately, in this way an extract concentration of 100 mg per mL was obtained. Afterward each stock extract was passed through 0.22 micron syringe filter to get a sterile solution. To prepare nutmeg extract dilutions, 0.5 mL of extract was taken in the sterile microtube, then diluted with distilled water at a ratio of 1:1, 1:2, 1:3,1:4, and 1:5. Similarly, dilution for myrobolan extract was done, which yielded a serially diluted concentration of 100, 50, 25, 12.5 and 6.25 mg/mL for each extract. Broth cultures of the pure culture isolates of *S. mutans* were prepared by transferring a loop of culture into the sterile nutrient broth and incubated at 37°C for 48 hours. From the prepared broth culture of pure bacterial stains, with the help of a sterile cotton swab inoculum was taken and seeded onto sterile Muller-Hinton agar plates and leaving it for 5 minutes before punching holes. After incubation, this will develop into diffused heavy lawn culture in the inoculated plates. To determine the antibacterial activity of the extracts well diffusion method was used which included the seed extract preparation using the standard procedure. The wells were then punched on agar plate with a diameter of 6 mm and stock extracts were taken into the micropipette and 20 µL of extract was discharged into the well, incubated at 37°C for 48 hours. Appearance of *zone of inhibition was seen* after incubation, *that is an area of an agar plate where the growth of an organism is prevented by nutmeg and myrobolan on the agar surface after the incubation period; it was measured using vernier caliper.* The minimum concentration at which the nutmeg and myrobolan extracts were achieved at 50% and 50%, respectively against *S.mutans*.

### Preparation of Gels

Swelling of gels (Carbapol, Guar juice, and Agar) were done for 24 hours in sterile distilled water. In a beaker containing 50 mL of sterile distilled water, swelled carbapol, guar juice, and agar were added and homogenized using stirrer. After homogenization, herbal powder was added and mixed properly. Now the volume of the mixture is made up to 100 mL and stirred to maintain the homogeneity. Then this mixture was placed in a beaker and after sealing, they were placed in the refrigerator. In final preparation, the carbapol was 5%, guar juice was 2%, and agar was 1%. So the herbal gel obtained was 2%.

### *In vivo* Procedure

Twenty participants were selected who reported to Department of Paedodontics and Preventive Dentistry. Healthy participants had bilateral involvement of primary molars were included in the study and were randomly (spin of a coin) assigned to the following groups:

- *Group 1: Myristica fragrans* (Nutmeg),
- *Group 2: Terminalia chebula* (Myrobolan),
- *Group 3:* Formocresol

### Inclusion Criteria

- Healthy periodontium.
- Restorable, at least 2/3rd of the root length
- No antibiotics should be received by the patient at least one week before the treatment.

### Exclusion Criteria

- Spontaneous/persistent pain
- Evidence of internal resorption
- Bone loss
- Abscess or fistula or teeth close to natural exfoliation were excluded from the study.^10^

In this study, split-mouth design performing an equal number of pulpotomies using herbal medicaments on one side and formocresol pulpotomy on the other side. The pulpotomy technique was standard 5 minutes pulpotomy on both the sides with additional 3 minutes application in case of uncontrolled bleeding.

### Procedure

Pulpotomy procedure was done on a total of 20 participants and 40 primary molars (2 molars per participant) under ideal conditions of sterilization and isolation with a rubber dam, and access cavity opening were done on the tooth selected. Routine armamentarium which is used during pulpotomy procedure was used along with freshly prepared Myristica *fragrans*, and *Terminalia chebula* gels. Spoon-excavator was used to remove coronal pulp. The pulp chamber was cleaned properly with normal saline, followed by hemostasis with wet cotton pellets and then gels were compressed against the amputated pulp for 5 minutes. If the hemostasis is not achieved then cotton pellet was compressed for another 3 minutes. The pulp chamber was then filled with zinc oxide eugenol (ZOE B&T; Caulk–Dentsply, Milford, DE, USA), and final restoration was done using glass ionomer, followed by stainless steel crown depending upon the need of the particular case.

Participants were recalled at the interval of 3, 6 and 12 months for clinical and radiographic evaluation by three experienced pediatric dentists who are blinded for the study. They were calibrated for the assessment of the radiographs and clinical examination by considering failure clinically and/or radiographically:

- One or more of the following signs were present: pain; swelling; mobility; percussion, pain; internal root resorption; and furcation and/or periapical bone destruction.
- Internal root resorption was regarded as a failure in all instances.

The data were assessed clinically, radiographically, and total success rate at each follow-up period.

The obtained data were analyzed using the Chi-square test with the level of significance at *p* < 0.05.

## RESULTS

Twenty children had a mean age at the time of initial treatment of 7.3 ± 1.2 years (range 6–9 years). Two children were excluded from the study who were not available at the 12-month follow-up. No significant difference between FC, nutmeg, and myrobolan for clinical and radiographic success rate at 3, 6 and 12 months (Tables 1 and 2). The overall clinical success rate for formocresol, nutmeg, and myrobolan at 3 months was 70, 60 and 80 and radiographic success rate was 90, 80, and 100, respectively (Table 3). At 3 months follow-up, furcal radiolucency was observed in two teeth in the FC group and two teeth in the nutmeg group. *At 3 months, cases failed either in clinical or in radiographic were excluded from 6 months clinical as well as radiographic analysis*. So, at 6 and 12 months follow-up, clinical success rates in the FC, nutmeg, and myrobolan groups were 100, 100 and 100, respectively, on the other hand, radiographic success rates were 100, 100 and 100, respectively for the three groups. There was no group showed internal root resorption appreciated in all the three groups at the end of 12 months.

## DISCUSSION

The controversies regarding the use of better treatment procedure and medicaments for vital molar pulpotomy are unsettled even now in the 21st century despite much impressive scientific advancement. Identifying the best amalgamation of ingredients and techniques to predictably produce pulpal healing after pulpotomy is still obscure. This leads to the propagation of herbal extracts in primary molar pulpotomies with minimal or no side effects.

**Table 1:** Clinical outcomes of formocresol, nutmeg and myrobolan at 3, 6 and 12 months follow-up

	*3 months* *FC* *N = 20*	*3 months* *nutmeg* *N = 10*	*3 months* *myrobolan* *N = 10*	*6 months* *FC* *N=14*	*6 months* *nutmeg* *N = 6*	*6 months* *myrobolan* *N = 8*	*12 months* *FC* *N = 14*	*12 months* *nutmeg* *N = 6*	*12 months* *myrobolan* *N = 8*
*Pain*	20 (100%)	8 (80%)	10 (100%)	14 (100%)	6 (100%)	8 (100%)	14 (100%)	6 (100%)	8 (100%)
*Sinus tract*	20 (100%)	10 (100%)	10 (100%)	14 (100%)	6 (100%)	8 (100%)	14 (100%)	6 (100%)	8 (100%)
*Swelling*	20 (100%)	8 (80%)	10 (100%)	14 (100%)	6 (100%)	8 (100%)	14 (100%)	6 (100%)	8 (100%)
*Mobility*	20 (100%)	8 (80%)	10 (100%)	14 (100%)	6 (100%)	8 (100%)	14 (100%)	6 (100%)	8 (100%)
*Percussion*	20 (100%)	8 (80%)	10 (100%)	14 (100%)	6 (100%)	8 (100%)	14 (100%)	6 (100%)	8 (100%)
*Restoration dislodgement*	14 (70%)	6 (60%)	8 (80%)	14 (100%)	6 (100%)	8 (100%)	14 (100%)	6 (100%)	8 (100%)
*Overall clinical success rate*	14 (70%)	06 (60%)	08 (80%)	14 (100%)	06 (100%)	8 (100%)	14 (100%)	06 (100%)	8 (100%)
*Chi-square test*	Yates’χ^2^ = 0.298, df = 2, *p* = 0.862 (>0.05) Not significant			Test not applicable as all samples in three groups showed success			Test not applicable as all samples in three groups showed success		

**Table 2:** Radiographic outcomes of formocresol, nutmeg and myrobolan at 3, 6 and 12 months follow-up

	*3 months* *FC* *N = 20*	*3 months* *nutmeg* *N = 10*	*3 months* *myrobolan* *N = 10*	*6 months* *FC* *N = 14*	*6 months* *nutmeg* *N = 6*	*6 months* *myrobolan* *N = 8*	*12 months* *FC* *N = 14*	*12 months* *nutmeg* *N = 6*	*12 months* *myrobolan* *N = 8*
*Furcal radiolucency*	18 (90%)	8 (80%)	10 (100%)	14 (100%)	6 (100%)	8 (100%)	14 (100%)	6 (100%)	8 (100%)
*Internal root resorption*	20 (100%)	10 (100%)	10 (100%)	14 (100%)	6 (100%)	8 (100%)	14 (100%)	6 (100%)	8 (100%)
*External root resorption*	20 (100%)	10 (100%)	10 (100%)	14 (100%)	6 (100%)	8 (100%)	14 (100%)	6 (100%)	8 (100%)
*Pulp canal obliteration*	20 (100%)	10 (100%)	10 (100%)	14 (100%)	6 (100%)	8 (100%)	14 (100%)	6 (100%)	8 (100%)
*Overall radiographic success rate*	18 (90%)	8 (80%)	10 (100%)	14 (100%)	06 (100%)	8 (100%)	14 (100%)	06 (100%)	8 (100%)
*Chi-square test*	Yates’χ^2^ = 0.694, df = 2, *p* = 0.707 (>0.05) Not significant			Test not applicable as all samples in three groups showed success			Test not applicable as all samples in three groups showed success		

**Table 3:** Overall success rate of formocresol, nutmeg and myrobolan at 3, 6 and 12 months

	*3months* *FC* *N = 20*	*3months* *nutmeg* *N = 10*	*3months* *myrobolan* *N =10*	*6months* *FC* *N =14*	*6months* *nutmeg* *N=6*	*6months* *myrobolan* *N = 8*	*12 months* *FC* *N = 14*	*12 months* *nutmeg* *N = 6*	*12 months* *myrobolan* *N = 8*
Overall success rate	14 (70%)	06 (60%)	08 (80%)	14 (100%)	06 (100%)	8 (100%)	14 (100%)	06 (100%)	8 (100%)

The present study is the randomized clinical trial on vital pulpotomy with herbal gels on human primary molar teeth. The standardization was achieved by randomly distributing the teeth among the treatment groups based on spin of the coin and each patient received pulpotomy with the two different medicaments. The split-mouth design was preferred so that the desired effects of herbal gels can be compared clinically and radiographically with formocresol on the same patient. Although, histological evaluation is considered a better test to predict pulpal healing followed by radiographical and clinical methods, but it needs extraction of teeth.^11^ Hence in the present study, only clinical and radiographic evaluation was utilized since teeth were destined for the preservation of its function in the oral cavity; so none of the teeth could be extracted and included for histological examination.

The ideal pulpotomy agent should accelerate the recovery of remaining radicular pulp tissue to a healthy state, so that involved tooth attains normal physiological state.^1^ The proof of the success of pulpotomy agents comes from clinical observation and experience. This led us to follow up the patients for clinical and radiographic outcomes for 3rd month, 6th month and 1 year. Two teeth reporting with pain was considered as a failure, the reasons may be dislodged restoration, but radiograph still reveals plug of gel over the radicular orifice with small amounts of temporary restorative material over it when looked upon clinically.

Moreover, a total of 14 teeth showed dislodged restorations over the period of 12 months which is not related to the failure of pulpotomy. Thus, failure was independent of timing and type of post-pulpotomy restoration placed. It can be interpreted that medicament itself rather than type and timing of restoration is responsible for the failure.

Formocresol is still considered the most popular devitalizing material in primary tooth pulpotomy. However, toxicity, mutagenicity, and carcinogenicity are still problematic features of FC, and an alternative is required.^12^ Formocresol is devitalizing agent whereas herbal gels like *Myristica fragrans* (MF)–nutmeg, *Terminalia chebula* (TC) maintain pulp vitality.

The chemical constituents of *Terminalia chebula* like chebulinic acid, tannin, galic acid, and ascorbic acids are the key factors for the efficient action of the species. Biochemical studies revealed that there is an increase in total protein, DNA, and collagen contents in the granulation tissues of treated wounds. The tensile strength of tissues in extract-treated incision wounds increased by about 40%. These results showed beneficial effects of *T. chebula* in the acceleration of the healing process.^13^

Essential oils obtained from *Myristica fragrans* seeds have growth inhibition capability against bacterial spores and can act as food preservatives.^14^ It has been observed experimentally that extract from the dried seed cover of *Myristica fragrans* contains two compounds and both exhibit strong antifungal and antibacterial activities. ^15^ In another study, it was found that ethyl acetate extract of the flesh of *Myristica fragrans* had strong bactericidal activity against some cariogenic Gram-positive and Gram-negative bacteria.^16^ The methanol extract from seeds of *Myristica fragrans* used for the treatment of inflammatory diseases also had inhibitory effects on nitric oxide (NO) production.^17^

Herbal gels have potential antibacterial activity, and thus it provides a scientific basis for the utilization of this plant in the treatment of the inflammatory process. Based on promising results of the study, further studies can be done for its use as an anti-inflammatory agent in an endodontic procedure like pulpotomy, as it is cheap and affordable for a common man.

## CONCLUSION

Herbal gels have a promising role in dentistry; having proper knowledge and their effects on teeth would prove them as a successful dental therapeutic agent. *Quite promising clinical and radiographic results of anti-inflammatory agents in the present study shows their potential to be an ideal pulpotomy agent. It becomes necessary to understand the biochemical interactions, characteristics, tissue uptake metabolism, protein synthesis, and the biological activities of various anti-inflammatory on pulpal healing. Understanding these biological interactions may provide a reliable biological method for vital pulp therapy of primary teeth and young permanent teeth in the clinical practice.*
